# Magnetic Fe_1−*x*_Zn_*x*_Fe_2_O_4_ nanoparticles as dual adsorbents for Cr(vi) and Direct Red 79: kinetics, isotherms, and mechanistic insights

**DOI:** 10.1039/d5ra07081c

**Published:** 2025-12-17

**Authors:** Vu Thi Hau, Nguyen Thuy Chinh, Pham Hoai Linh, Nguyen Thi To Loan, Ngo Thi Mai Viet, Dang Duc Dung, Nguyen Quoc Dung

**Affiliations:** a Faculty of Chemistry, Thai Nguyen University of Education 20 Luong Ngoc Quyen Thai Nguyen Vietnam dungnq@tnue.edu.vn; b Institute of Materials Science, Vietnam Academy of Science and Technology 18 Hoang Quoc Viet, Nghia Do Ha Noi Vietnam; c Multifunctional Ferroics Materials Lab., Faculty of Engineering Physics, Ha Noi University of Science and Technology 1 Dai Co Viet Road Ha Noi Vietnam

## Abstract

Magnetic spinel ferrites are attractive adsorbents for complex wastewaters because they couple high affinity with rapid magnetic recovery. Here, a series of Zn-substituted ferrites (Fe_1−*x*_Zn_*x*_Fe_2_O_4_, *x* = 0.0–1.0) was synthesized by co-precipitation and systematically characterized. The series samples display particles in the nanoscale range from 8–50 nm, single phase with spinel structure, tunable magnetization, and mesoporosity where the highest surface area of 228 m^2^ g^−1^ were estimated for Fe_0.4_Zn_0.6_Fe_2_O_4_ samples. The point of zero charge is obtained around 6.26, consistent with strong uptake of anionic species under mildly acidic conditions. Batch adsorption toward an organic dye (Direct Red 79, DR79) and an inorganic oxyanion (hexavalent chromium, Cr(vi)) shows optimal removal at pH = 3, with equilibrium contact times of ∼120 min for DR79 and ∼90 min for Cr(vi). Nonlinear kinetic fitting indicates Elovich behaviour for DR79 (heterogeneous chemisorption with site-energy distribution) and pseudo-second-order kinetics for Cr(vi). Intraparticle diffusion contributes to the rate of reaction but is not rate-limiting. Nonlinear isotherm analysis indicates that Freundlich is applicable to both solutes, while Langmuir capacities reach approximately 95.3 mg g^−1^ and 62.1 mg g^−1^ for DR79 and Cr(vi), respectively. Thermodynamic analysis reveals spontaneous adsorption in all cases. The uptake of DR79 is endothermic (Δ*H*° ≈ +79.1 kJ mol^−1^; Δ*G*° ≈ −4.5 to −8.5 kJ mol^−1^; Δ*S*° ≈ +0.271 kJ mol^−1^ K^−1^), whereas Cr(vi) is exothermic (Δ*H*° ≈ −40.0 kJ mol^−1^; Δ*G*° ≈ −2.0 to −0.15 kJ mol^−1^; Δ*S*° ≈ −0.126 kJ mol^−1^ K^−1^). The results highlight Fe_0.4_Zn_0.6_Fe_2_O_4_ composition displayed as a magnetically retrievable, dual-function adsorbent capable of treating mixed organic/inorganic contaminants with reliable nonlinear model parameterization for process design.

## Abstract 2

Magnetic spinel ferrites are attractive adsorbents for complex wastewaters because they couple high affinity with rapid magnetic recovery.

## Graphical abstract

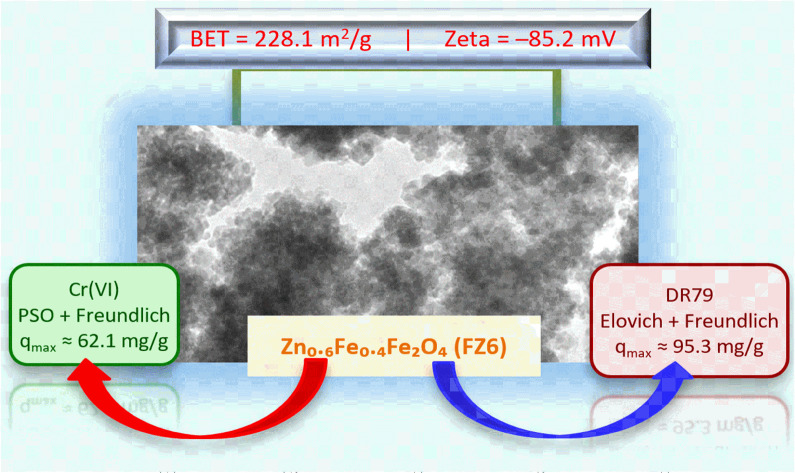

## 1. Introduction

Industrial wastewater is characterised by a complex mixture of organic dyes and toxic heavy metals, which pose a significant threat to ecosystems and human health. Azo dyes used in textiles (*e.g.* Direct Red 79) and heavy metals like hexavalent chromium (Cr(vi)) are among the most problematic pollutants.^1–4^ Direct Red 79 (DR79) is an azo dye extensively used in textile, paper, and leather processing; improper disposal of DR79 leads to persistent contamination of water bodies because it resists biodegradation.^1,5^ Moreover, DR79 and related benzidine-based dyes are of health concern – they can metabolize into carcinogenic aromatic amines (*e.g.* benzidine), raising toxicity issues in the environment. Similarly, Cr(vi) is a highly toxic heavy metal (far more toxic and mobile than Cr(iii)) and is classified as a known carcinogen; even trace levels in water above regulatory limits (0.05–0.1 mg L^−1^) can cause severe health problems including organ damage and cancer.^2^ Major sources of Cr(vi) include effluents from electroplating, leather tanning, dyes/pigments, and other industrial processes.^2,6–9^ The persistence and toxicity of such pollutants demand effective remediation strategies before wastewater is discharged.

Conventional treatment methods for dyes and heavy metals (such as chemical precipitation, redox reactions, membrane filtration, and advanced oxidation) often suffer from high cost or generate secondary waste.^2,10,11^ In contrast, adsorption has emerged as a simple, safe, and economical approach for removing both dyes and metal ions from contaminated water.^12,13^ The adsorption process is highly flexible and can achieve high removal efficiencies without producing harmful by-products. A wide variety of adsorbent materials – from activated carbons and clays to novel nanomaterials – have been investigated, many of which offer high capacity and reusability.^13^ In particular, *in situ* adsorption using solid sorbents is attractive for its operational simplicity and the ease of incorporating adsorbents into water treatment systems.

Nanoscale adsorbents are especially effective due to their large surface area and tunable surface chemistry.^14^ Among these, magnetic iron oxides and ferrites have gained considerable attention for water treatment.^1,15^ Ferrites are iron oxide-based magnetic compounds that can be quickly recovered after use by applying an external magnetic field. This feature is a key advantage in wastewater treatment, as it allows for facile separation of the spent adsorbent without filtration or centrifugation. For example, zinc ferrite (ZnFe_2_O_4_) is a magnetic spinel that has been widely studied for pollutant removal due to its chemical stability, non-toxicity, low cost, and insolubility in water.^12^ Unlike pure magnetite (Fe_3_O_4_, referred as Fe_1_^2+^Fe_2_^3+^O_4_) which can undergo oxidation or phase changes over time, spinel ferrites like ZnFe_2_O_4_ exhibit high phase stability and corrosion resistance.^12^ They also remain stable in acidic conditions (pH = 2–6), where many metal-oxide adsorbents lose capacity, making them suitable for treating acidic effluents.^16,17^ Furthermore, the strong magnetization of such materials enables easy recovery and reuse; studies have shown that magnetically separable nano-adsorbents can be regenerated multiple times, maintaining significant removal efficiency over successive cycles.^18^

Doped or substituted spinel ferrites of the form Fe_1−*x*_M_*x*_Fe_2_O_4_ (where M is a divalent metal) allow tuning of the adsorbent's properties.^1^ Partial substitution of Fe^2+^ with other cations (*e.g.* Co^2+^, Zn^2+^, Mn^2+^) can modify the surface chemistry and magnetic behavior of the ferrite, potentially enhancing adsorption performance. In particular, zinc-substituted magnetite (Fe_1−*x*_Zn_*x*_Fe_2_O_4_) offers a way to adjust the Fe^2+^/Fe^3+^ ratio and magnetization of the particles.^19^ Prior work on analogous ferrites has shown improved dye adsorption at certain doping levels – for instance, Fe_1−*x*_Co_*x*_Fe_2_O_4_ with *x* ≈ 0.5 exhibited optimal uptake of Direct Red 79 at low pH.^1^ Zinc ferrite and its composites have likewise demonstrated effective removal of organic dyes: ZnFe_2_O_4_ on reduced graphene oxide removed ∼98% of methylene blue in 30 minutes under optimal conditions,^12^ and pure ZnFe_2_O_4_ achieved high uptake of anionic dyes like Congo Red at acidic pH (*e.g.* >90% removal at pH ∼ 3.5).^12^ However, there have been comparatively fewer studies focusing on a single magnetic nanoadsorbent that can address both heavy metal anions and organic dyes simultaneously. Deploying a dual-function adsorbent for different classes of pollutants is desirable for integrated wastewater treatment, provided it maintains high affinity for both types of contaminants.

To design an efficient adsorption system, it is essential to understand the kinetics and equilibrium behavior of pollutant uptake. Adsorption kinetics reveal how quickly contaminants can be sequestered and often provide insight into the rate-limiting steps (film diffusion, pore diffusion, surface re action, *etc.*). Many dye and heavy metal adsorption processes follow a pseudo-second-order kinetic model, suggesting chemisorption as the rate-controlling mechanism.^20^ For instance, studies on similar ferrite nanoadsorbents reported that Direct Red 79 removal obeyed pseudo-second-order kinetics, with intraparticle (intragranular) diffusion also contributing to the overall rate.^1^ In the case of Cr(vi) adsorption, the uptake rate is often rapid initially and fits well to pseudo-second-order kinetics as well, indicating strong affinity and possible chemical interactions.^2^ Equilibrium isotherm analysis is equally important: isotherm models such as Langmuir, Freundlich, Temkin, and Redlich–Peterson are commonly applied to describe how pollutants partition between solution and solid phases at equilibrium.^1^ A Langmuir model assumes monolayer adsorption on a homogeneous surface, while Freundlich and Temkin models account for heterogeneous surface energies and adsorbate–adsorbent interactions. In recent years, researchers have favored non-linear regression methods to determine isotherm and kinetic parameters, rather than linearizing these models. Non-linear fitting avoids the bias and error amplification that can arise from linear transformations of models.^21^ Indeed, it has been shown that non-linear regression yields more accurate isotherm constants and better reflects the true adsorption capacity.^20,21^ In this work, we adopt non-linear analysis of adsorption data to obtain reliable kinetic and isotherm parameters, ensuring robust evaluation of the adsorption performance.

Analyzing adsorption thermodynamics provides deeper insight into the nature of the sorption process. The key thermodynamic parameters that are to be considered are the change in Gibbs free energy (Δ*G*°), enthalpy (Δ*H*°), and entropy (Δ*S*°) for adsorption. The spontaneous adsorption process is indicated by negative values of the free energy change (Δ*G*°), and the magnitude of the enthalpy change Δ*H*° can distinguish physisorption from chemisorption.^1^ In general terms, a, Δ*H*° of less than 20–40 kJ mol^−1^ is indicative of physisorption, whereby weak forces are the governing forces. Conversely, a Δ*H*° in the order of 40–200 kJ mol^−1^ implies chemisorption, involving stronger bonding or possibly surface reactions.^1^ Temperature-dependent equilibrium studies allow calculation of these parameters. For example, adsorption of DR79 on cobalt-doped ferrite was found to be spontaneous (Δ*G*° ∼ −5 to −11 kJ mol^−1^ over 303–323 K) and strongly endothermic (Δ*H*° ≈ 74.9 kJ mol^−1^), indicating a chemisorption mechanism.^1^ Similarly, Cr(vi) adsorption is often enhanced at higher temperatures, which manifests as positive Δ*H*° (endothermic uptake) and increased adsorption capacity with temperature.^2^ Besides thermodynamics, mechanistic studies are vital to confirm how contaminants are bound or transformed by the adsorbent. In the case of Cr(vi), iron-based adsorbents can not only attract the anionic Cr(vi) species (such as HCrO_4_^−^) electrostatically but also chemically reduce Cr(vi) to the far less toxic Cr(iii) form.^22^ X-ray photoelectron spectroscopy (XPS) and other analyses have indeed confirmed the reduction of Cr(vi) to Cr(iii) on Fe-based magnetic sorbents, implicating surface Fe^2+^ as the electron donor in the redox adsorption mechanism.^22^ The resulting Cr(iii) may remain immobilized on the nanoparticle surface (as Cr(iii) complexes or precipitates), effectively removing it from solution In the case of organic dyes such as DR79, the proposed adsorption mechanisms generally involve electrostatic attraction between the negatively charged sulfonate groups of the dye and the positively charged adsorbent surface (at an acidic pH), as well as the potential for π–π interactions or hydrogen bonding with surface functional groups.^5^ Changes in the adsorbent's FTIR spectra and zeta potential before *vs.* after dye uptake can provide evidence for these interactions.^5^ Furthermore, the reusability of the adsorbent is an important practical consideration tied to mechanism: a robust adsorbent should retain its structure and active sites over multiple adsorption–desorption cycles. Magnetic ferrite nanoparticles can be regenerated and reused, though some loss of capacity is expected after repeated cycles.^1^ For instance, Zn/Co-doped ferrite nanoparticles retained a majority of their dye removal efficiency after a few reuses, until active site saturation gradually reduced performance.^1^ This highlights the balance between strong binding (good for high initial uptake) and ease of desorption (good for complete regeneration).

Nanoparticles as dual adsorbents for Cr(vi) and Direct Red 79 are aimed at developing a single magnetic nanoadsorbent capable of removing both an inorganic contaminant (Cr(vi)) and an organic dye (DR79) from water. In this work, Zn-substituted iron ferrite nanoparticles were synthesized and characterized, and their dual adsorption performance was evaluated. Batch adsorption experiments were conducted to measure removal efficiencies under varying conditions, and the data were analyzed with multiple kinetic models (pseudo-first-order, pseudo-second-order, intraparticle diffusion) and isotherm models (Langmuir, Freundlich, *etc.*). Non-linear regression was employed to fit the models for accurate parameter determination, following best practices in adsorption modeling.^21^ Thermodynamic parameters (including of Δ*G*°, Δ*H*°, Δ*S*°) were calculated to determine the spontaneity and heat changes of the adsorption of Cr(vi) and DR79. Finally, we investigated the adsorption mechanisms for each pollutant by examining factors such as pH effects, potential redox interactions, and changes in the adsorbent's properties after uptake. By focusing on both magnetic retrievability and broad-spectrum adsorption capability, this study provides insight into the design of versatile nanoadsorbents for comprehensive wastewater remediation.^1,12^ The findings provide a mechanistic understanding of the application of magnetic Fe_1−*x*_Zn_*x*_Fe_2_O_4_ in the treatment of wastewater contaminated with a mixture of organic and inorganic pollutants. In addition, they offer practical guidance on its utilisation.

## 2. Experiment

### 2.1. Chemicals and instruments

Iron(ii) chloride tetrahydrate (FeCl_2_·4H_2_O, ≥99.0%), iron(iii) chloride hexahydrate (FeCl_3_·6H_2_O, ≥99%), zinc chloride (ZnCl_2_·9H_2_O, ≥98%), and sodium hydroxide (NaOH, ≥98% anhydrous) powdered chemicals were purchased from Merck. Direct Red 79 (DR79, C_37_H_28_N_6_Na_4_O_17_S_4_, ≥98%), hydrochloric acid (HCl, 37% solution) and acetone (CH_3_COCH_3_, ≥99.5%) were purchased from China. Solutions of 2 M FeCl_3_, 2 M FeCl_2_, 2 M ZnCl_2_, 2 M HCl, and 2 M NaOH were prepared from stock chemicals using twice-distilled water. A magnetic heating stirrer (C-MAG HS, IKA) was used for the synthesis of materials. IKA®KS 260 basic and control shakers were used to study adsorption. The adjustment of the pH of the solution containing the adsorbate was achieved by means of a pH meter.

### 2.2. Fabrication of Fe_1−*x*_Zn_*x*_Fe_2_O_4_

In the synthesis of Fe_1_Fe_2_O_4_ nanoparticles, a mixture comprising 2 mL of 2 M FeCl_2_ and 4 mL of 2 M FeCl_3_ (resulting in 8 mL of 1 M FeCl_3_) was combined with 10 mL of 2 M HCl. Next, 60 mL of 2 M NaOH was heated to 100 °C and the iron salt solution was quickly added to the boiling NaOH. The iron salt was poured in quickly to ensure uniform nucleation with the NaOH. Following the reaction, the mixture was filtered, followed by two washes with distilled water until a pH of 7 was reached. The final step involved rinsing the product in acetone.

The fabrication process of Fe_1−*x*_Zn_*x*_Fe_2_O_4_ was used to similar to that of Fe_1_Fe_2_O_4_ nanoparticles. However, instead of using FeCl_2_, a portion of the mixed solution was replaced by ZnCl_2_ in a certain ratio, as shown in Table S1. The pure ferrite sample (Fe_1_Fe_2_O_4_) and zinc-doped ferrite (Fe_1−*x*_Zn_*x*_Fe_2_O_4_) samples with Zn concentrations of 20%, 40%, 50%, 60%, 80%, and 100% were prepared and labeled as FZ0, FZ2, FZ4, FZ5, FZ6, FZ8, and FZ10, respectively.

### 2.3. Morphology and structures characterization

The crystal and phase structure, morphology, particle size, and chemical structure of the synthesized samples were characterized by field-emission scanning electron microscopy (SEM) system (S-4800, Hitachi), X-ray diffraction (XRD) analysis (XRD-Bruker D8 Advance), Fourier transform infrared spectroscopy (FTIR; Nicolet Nexus 670), and Ultraviolet-visible diffuse reflectance spectroscopy (UV-Vis-DRS; Hitachi U-2900). Magnetic properties were measured using a VSM, with results presented in magnetization plots. The specific surface area and pore size distribution was determined through nitrogen adsorption measurements at a low temperature (77.35 K) by using the Brunauer–Emmett–Teller (BET) method, with analysis performed using MicroActive for TriStar II Plus 2.03.

### 2.4. Adsorption properties

The concentration of adsorbate (DR79) was determined using a UV-1700 spectrophotometer (Shimadzu, Japan). The adsorption efficiency (removal percentage, *R*(%)) and the adsorption capacity at time *t* (denoted as *q*_*t*_) were calculated using the following equations:
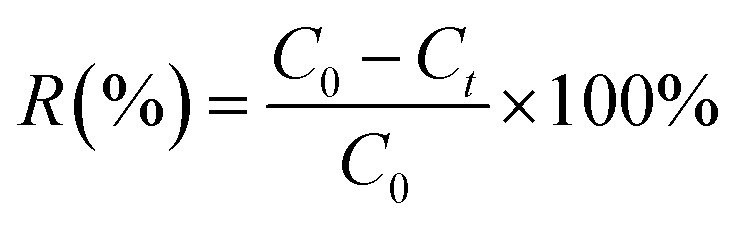

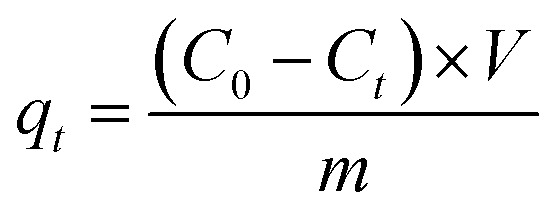


To investigate the adsorption mechanisms, the experimental kinetic data were fitted using the following nonlinear kinetic models:

Pseudo-first-order model (PFO):*q*_*t*_ = *q*_e_(1 − e^−*k*_1_*t*^)

Pseudo-second-order (PSO):
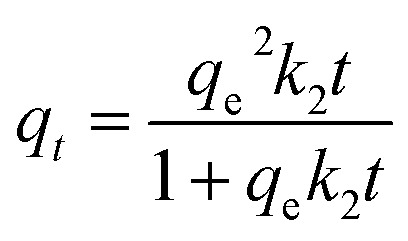


Elovich:
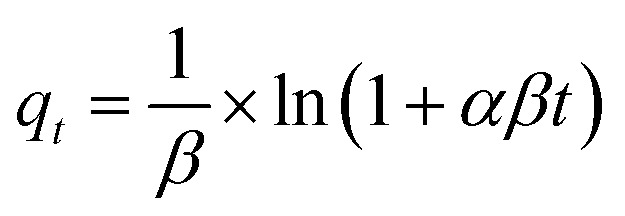


Intraparticle diffusion (Weber–Morris):*q*_*t*_ = *k*_id_ × *t*^1/2^ + *C*where *q*_e_ is the adsorption capacity at equilibrium (mg g^−1^); *k*_1_ (min^−1^) and *k*_2_ (g mg^−1^ min^−1^) are the rate constants for PFO and PSO models, respectively; *α* (mg g^−1^ min^−1^) and *β* (g mg^−1^) are the Elovich constants; *k*_id_ (mg g^−1^ min^−0.5^) is the intraparticle diffusion rate constant and *C* (mg g^−1^) indicates the boundary layer thickness.

The fitting quality of each model was assessed using the coefficient of determination *R*^2^, root mean square error (RMSE), and the consistency of calculated *q*_e_ with experimental values.

Equilibrium adsorption data were further fitted using nonlinear forms of classical isotherm models.

Where *q*_m_ (mg g^−1^) is the maximum adsorption capacity, *R* is the universal gas constant (8.314 J mol^−1^ K^−1^), and *T* is the absolute temperature (K).

Langmuir isotherm:
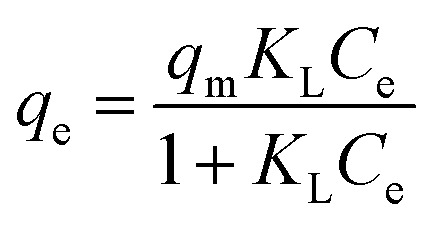
*K*_L_ (L mg^−1^) is the Langmuir constant.

Freundlich isotherm:*q*_e_ = *K*_F_*C*^1/*n*^_e_*K*_F_ and *n* are Freundlich constants related to adsorption capacity and intensity.

Temkin isotherm:
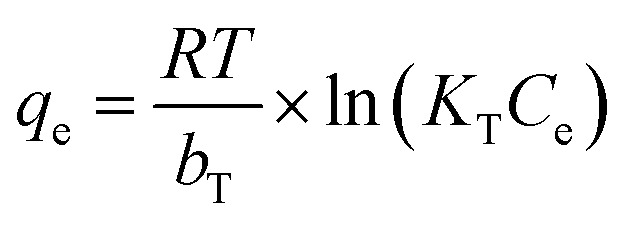
*K*_T_ (L g^−1^) and *b*_T_ (J mol^−1^) are Temkin constants associated with equilibrium binding and heat of adsorption, respectively.

Dubinin–Radushkevich (D–R) isotherm:*q*_e_ = *q*_m_ × e^−*Bε*^2^^with 
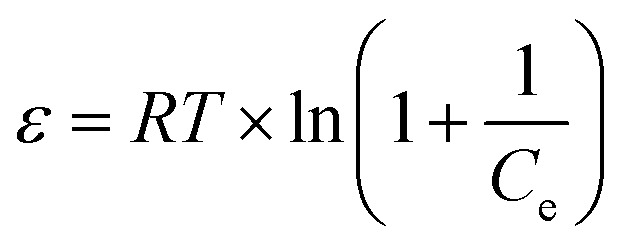
, *E* is mean adsorption energy 
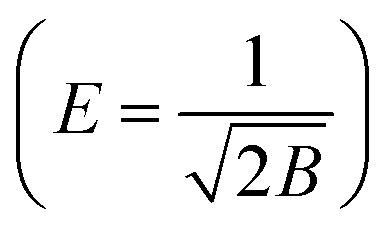
; *q*_m_ is theoretical saturation capacity (mg g^−1^); *B* is D–R constant related to mean adsorption energy (mol^2^ kJ^−2^); and *ε* is Polanyi adsorption potential (J mol^−1^).

## 3. Results and discussion

### 3.1. Morphology and structure of materials

**Fig. 1:**
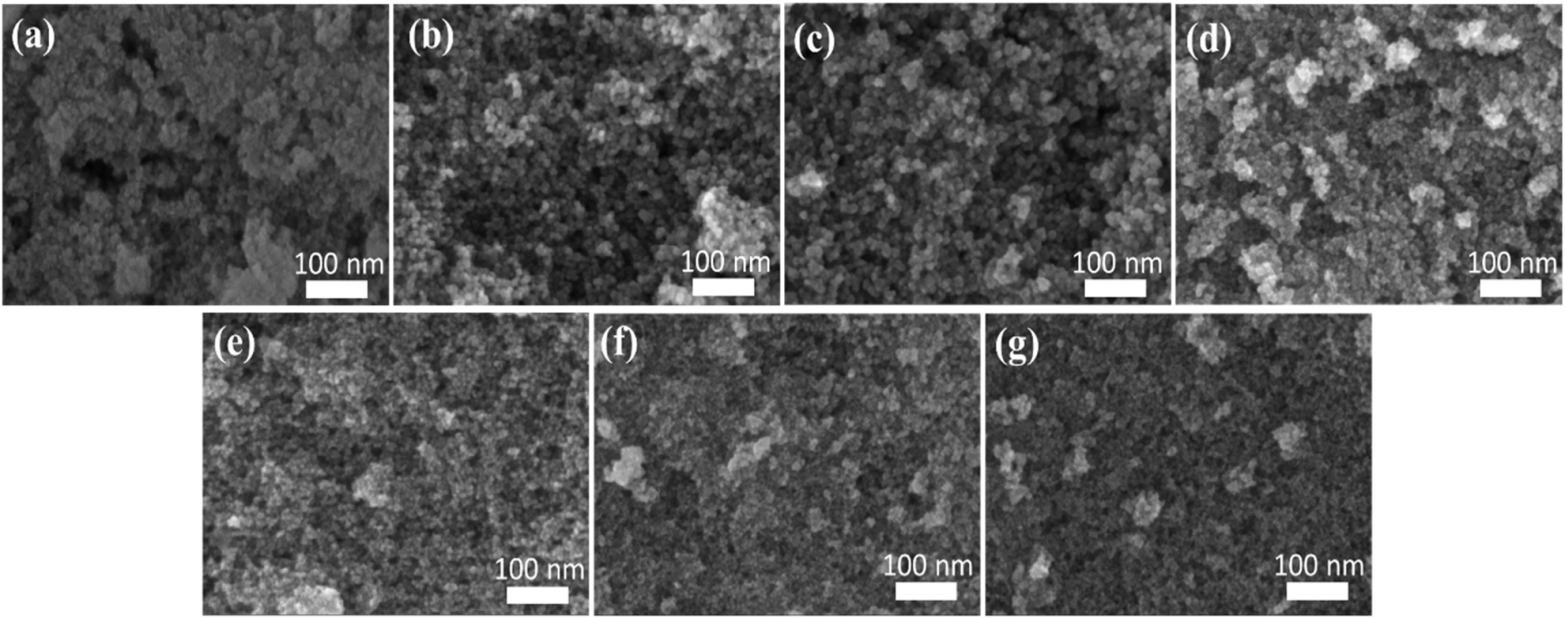
FE-SEM images of Fe_1−*x*_Zn_*x*_Fe_2_O_4_ nanoparticles with varying Zn^2+^ substitution ratios: (a) FZ0, (b) FZ2, (c) FZ4, (d) FZ5, (e) FZ6, (f) FZ8, and (g) FZ10.

Fig. 1 shows the FE-SEM micrographs of the FZ series samples from FZ0 to FZ10. All samples display relatively uniform nanostructures, with primary particle sizes predominantly around 10 nm. The surfaces appear rough and composed of aggregated spherical or quasi-spherical nanoparticles. A detailed comparison indicates a morphological evolution with increasing Zn^2+^ substitution. FZ0 (a) shows densely packed small grains with less obvious boundaries, suggesting high nucleation but limited crystal growth. As the Zn^2+^ content increases (FZ2 to FZ6), the particles become more distinct with clearer contours, suggesting an enhanced grain growth or better crystallinity due to the substitutional effect of Zn^2+^ in the spinel lattice. FZ5 and FZ6 (d and e) represent optimal morphology with homogeneous and well-dispersed nanoparticles, indicating a balance between nucleation and growth processes. For FZ8 and FZ10 (f and g), some degree of aggregation is observed, possibly due to increased surface energy or magnetic interaction between particles. Overall, the FE-SEM images confirm the formation of nanosized particles with good dispersion and that Zn^2+^ substitution effectively influences the microstructural evolution of the synthesized ferrite materials. Nanoscale morphology is beneficial for adsorption applications due to the high surface area and abundant active sites.

**Fig. 2:**
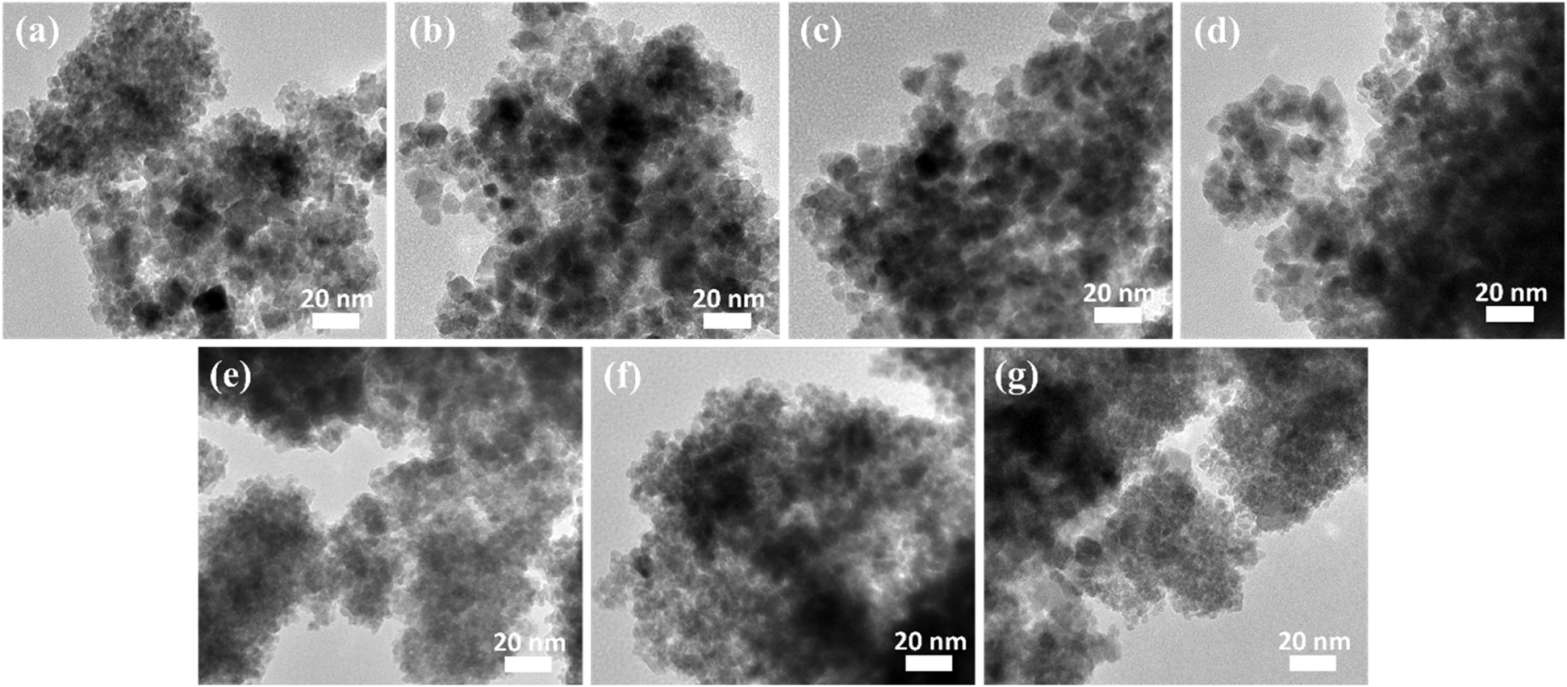
TEM images of (a) FZ0, (b) FZ2, (c) FZ4, (d) FZ5, (e) FZ6, (f) FZ8, and (g) FZ10.

Transmission electron microscopy (TEM) images further confirm the nanoscale morphology of the Fe_1−*x*_Zn_*x*_Fe_2_O_4_ nanoparticles as shown in Fig. 2(a–g) for FZ0 to FZ10 samples, respectively. FZ0 and FZ2 (Fig. 2(a and b)) show relatively more agglomerated structures, suggesting stronger interparticle interactions possibly due to limited zinc substitution. Across all samples (FZ0 to FZ10), the particles appear as loosely aggregated clusters composed of fine, quasi-spherical nanocrystallites with typical sizes in the range of 8–10 nm. As the Zn^2+^ content increases (FZ4–FZ6, Fig. 2(c–e)), the particles appear better dispersed, with slightly reduced agglomeration. This suggests that Zn^2+^ substitution may disrupt the magnetic interactions between Fe sites, reducing aggregation. FZ8 and FZ10 (Fig. 2(f and g)) still maintain nanoscale morphology but show increased particle overlapping and darker contrast, which may indicate either increased particle size or stacking of multiple nanocrystallites.

**Fig. 3:**
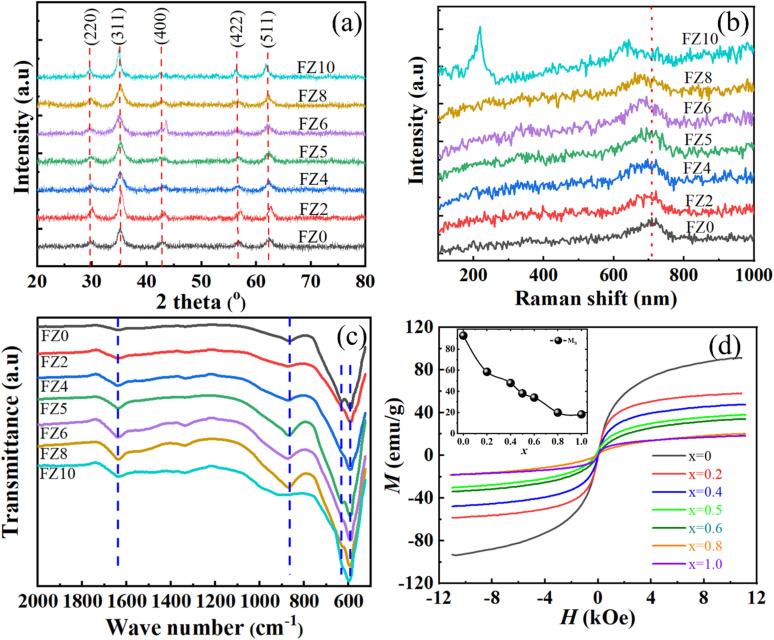
(a) XRD patterns, (b) Raman spectra, (c) FTIR spectra, and (d) magnetic hysteresis loops at room temperature.

The XRD patterns of the synthesized Fe_1−*x*_Zn_*x*_Fe_2_O_4_ (FZ0–FZ10) samples are shown in Fig. 3(a). All samples exhibit characteristic diffraction peaks at 2*θ* ≈ 30.1°, 35.5°, 43.1°, 53.5°, 57.0°, and 62.6°, which correspond to the (220), (311), (400), (422), (511), and (440) planes of the cubic spinel structure, respectively, and are in good agreement with the standard JCPDS card no. 22-1086 for Fe_1_Fe_2_O_4_ crystal structure. This confirms the formation of a single-phase spinel structure. With increasing Zn^2+^ content, the diffraction peaks slightly shift toward lower angles, indicating lattice expansion due to the substitution of Fe^2+^ (ionic radius ∼0.78 Å) with larger Zn^2+^ ions (ionic radius ∼0.82 Å). The observed distortion in the lattice parameters of the Fe_1_Fe_2_O_4_ samples as a function of Zn dopant concentration supports the random distribution of Zn ions within the host lattice of Fe_1_Fe_2_O_4_ crystals. Furthermore, the evidence of the random substitution of Zn^2+^ cations into the host lattice of Fe_1_Fe_2_O_4_ crystals was supported by Raman spectroscopy, where the presence of Zn^2+^ cations altered the characteristic vibrational modes of Fe_1_Fe_2_O_4_.

In the Raman spectra (Fig. 3(b)), all samples exhibit a prominent peak in the region of ∼675–680 cm^−1^, which corresponds to the A_1g_ symmetric stretching vibration mode of the metal–oxygen (M–O) bond in the tetrahedral sites of the spinel lattice. As the Zn^2+^ content increases from FZ0 to FZ10, a noticeable red shift of this peak is observed accompanied by a progressive broadening of the peak profile. This shift toward lower wavenumbers indicates a structural change resulting from the substitution of Fe^2+^ ions by Zn^2+^ ions at the tetrahedral sites. Since Zn^2+^ has a larger ionic radius (0.82 Å) than Fe^2+^ (0.77 Å) and forms weaker Zn–O bonds, the replacement reduces the vibrational frequencies, thereby accounting for the observed peak shift and broadening. Additionally, the incorporation of nonmagnetic Zn^2+^ weakens the superexchange interactions between tetrahedral and octahedral sites, leading to decreased lattice stiffness. The broadened and red-shifted Raman peak also reflects increased cation redistribution and local structural disorder within the spinel lattice. These observations provide further evidence for the successful formation of a Fe_1−*x*_Zn_*x*_Fe_2_O_4_ solid solution structure and are consistent with the phase evolution revealed by XRD analysis. The absence of secondary ZnO diffraction peaks in the XRD patterns confirms that Zn^2+^ ions are incorporated into the spinel lattice rather than forming a separate ZnO phase.

In addition, the random distribution of Zn^2+^ cations into the host Fe_1_Fe_2_O_4_ crystal structure was investigated using Fourier Transform Infrared (FTIR) spectroscopy. The substitution of Fe^2+^ by Zn^2+^ led to modifications in the Fe–O vibrational modes, indicating changes in the bonding environment due to Zn incorporation. The effect of Zn^2+^ ion substitution on the chemical structure of Fe_1−*x*_Zn_*x*_Fe_2_O_4_ (FZ0–FZ10) samples was investigated through Fourier transform infrared spectra (Fig. 3(c)). The IR absorption bands of the spinel ferrite at low wavenumber related to the vibrational groups of metal–oxygen bonds.^23^ Although the spectrum was run from 500 cm^−1^ to 4000 cm^−1^, the bands related to metal-oxide bonds below 1200 cm^−1^ are considered. The bands with peaks at 635 and 590 cm^−1^ are the characteristic absorption bands of Fe–O bonds in Fe_1_Fe_2_O_4_ nanoparticles.^24,25^ The bands with peaks at 635 and 590 cm^−1^ correspond to the stretching vibrations of Fe^3+^–O bonds and Fe^2+^–O bonds in tetrahedral sites (A-sites). Compared with the pristine Fe_1_Fe_2_O_4_ nanoparticles, the intensity and peak positions of Fe–O bond sites have a slight shift and become narrowed when increasing the concentration of Zn^2+^ ion substitution. This evidence shows a change in the chemical structure of Fe_1−*x*_Zn_*x*_Fe_2_O_4_ samples, demonstrating that Fe^2+^ ions are substituted by Zn^2+^ cations at *A*-sites in the crystal lattice of Fe_1_Fe_2_O_4_ lead to change in crystalline field effect and strain in lattice by the increase of Zn^2+^ amounts.

Indirect evidence of the replacement of Fe^2+^ cations in the host lattice by Zn^2+^ cations indicated a significant effect on the magnetic properties of the Fe_1_Fe_2_O_4_ material. Magnetic hysteresis loops (Fig. 3(c)) at room temperature confirm the ferromagnetic nature of the synthesized nanoparticles, with a notable decrease in saturation magnetization (*M*_S_) values as Zn^2+^ concentration increases. Pure Fe_1_Fe_2_O_4_ exhibits the highest saturation magnetization value, approximately 92.8 emu g^−1^. This value decreases monotonically with increasing Zn^2+^ substitution, reaching around 18.2 emu g^−1^ for ZnFe_2_O_4_ (ZF10 sample). The reduction in *M*_S_ values correlate directly with Zn^2+^ substitution, indicating that Zn^2+^ ions replace Fe^2+^ in the tetrahedral sites. This substitute consequently dilutes the magnetic interactions, as Zn^2+^ cations are non-magnetic and do not contribute to the overall magnetic moment.

**Fig. 4:**
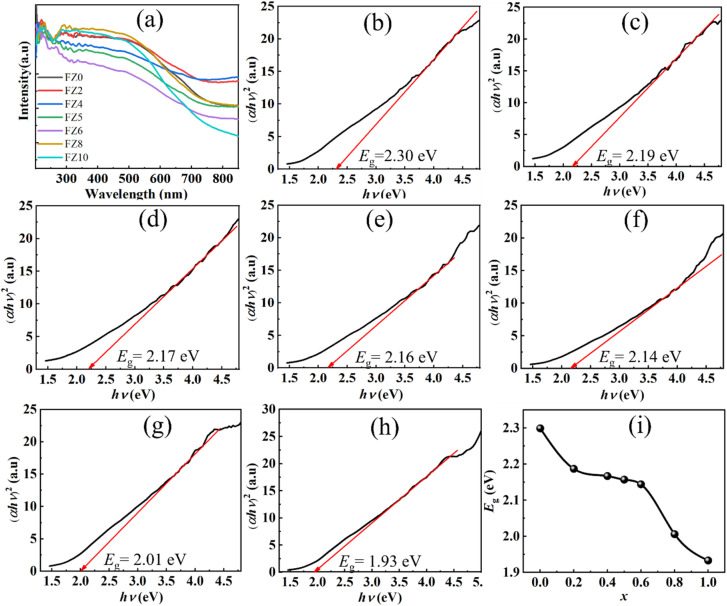
(a) UV-Vis diffuse reflectance spectra (DRS) of Fe_1−*x*_Zn_*x*_Fe_2_O_4_ samples as a function of Zn^2+^ doping concentration, Tauc plots used for optical band gap determination of individual samples: (b) FZ2, (c) FZ4, (d) FZ5, (e) FZ6, (f) FZ8, and (g) FZ10, and (i) variation in optical band gap energy of Fe_1_Fe_2_O_4_ with increasing Zn^2+^ content.

Fig. 4(a) presents the UV-Vis diffuse reflectance spectra (DRS) of the Zn^2+^-doped Fe_1_Fe_2_O_4_ samples with various of Zn amounts. All samples exhibited strong absorption in visible and near-infrared regions, characteristic of Fe_1_Fe_2_O_4_-based materials. With increasing Zn^2+^ content, the absorption edge gradually shifted toward longer wavelengths (red-shift), indicating a narrowing of the optical band gap. This red-shift can be attributed to the substitution of Fe^2+^/Fe^3+^ of host Fe_1_Fe_2_O_4_ crystal structural by Zn^2+^ ions, which alters the electronic structure and induces lattice distortions, thereby generating defect states within the band gap. The Tauc plots, (*αhv*)^2^ as function of absorption photon energy (*hv*), in Fig. 4(b–h) was used to determine the direct optical band gap energies (*E*_g_), revealing a progressive decrease from approximately 2.299 eV for FZ0 to 1.933 eV for FZ10. As shown in Fig. 4(i), the reduction in *E*_g_ values follow a non-linear trend, with a slow decrease for Zn dopant concentration below 40 mol% and a more pronounced drop for Zn dopant amounts over 80 mol%, possibly due to the solubility limit of Zn^2+^ in the Fe_1_Fe_2_O_4_ lattice and the associated structural distortions. The narrowed band gap enhances visible-light absorption and may promote surface activation under ambient light. In other words, the substitution of Zn^2+^ cations into the host Fe_1_Fe_2_O_4_ crystal significantly affected the crystal structure and strongly modified the properties of the host material.

**Fig. 5:**
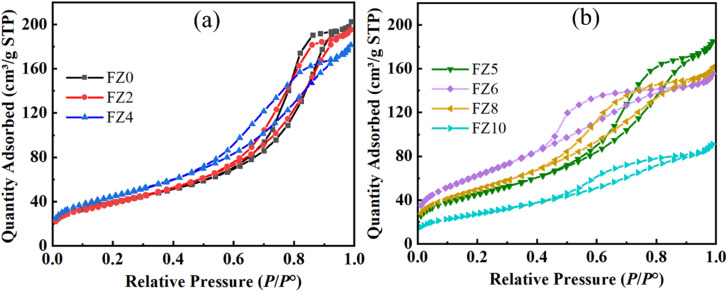
Nitrogen adsorption–desorption isotherms of Fe_1−*x*_Zn_*x*_Fe_2_O_4_ samples with different Zn^2+^ substitution levels: (a) FZ0, FZ2, and FZ4; (b) FZ5, FZ6, FZ8, and FZ10.

For further characterization of the absorbance properties of Zn-modified Fe_1_Fe_2_O_4_ samples, surface area and pore volume are important parameters that must be optimized prior to evaluating their adsorption performance for Direct Red 79 (DR79). The nitrogen adsorption–desorption isotherms presented in Fig. 5(a and b) exhibit typical type IV behavior with H3-type hysteresis loops, confirming the presence of mesoporous structures. This mesoporosity is attributed to interparticle voids originating from the aggregated Fe_1−*x*_Zn_*x*_Fe_2_O_4_ nanoparticles, in agreement with the morphology observed in the TEM images.

Table 1 presents detailed BET surface area and pore characteristics, showing that the BET surface area significantly increases with Zn content, peaking at 228.109 m^2^ g^−1^ for sample FZ6, before declining at higher Zn concentrations. The increase in surface area is consistent with decreased particle and pore size, suggesting enhanced porosity and higher active sites availability for adsorption.

**Table 1:** Textural properties of the synthesized Fe_1−*x*_Zn_*x*_Fe_2_O_4_ nanoparticles obtained from BET analysis

Samples	BET area (m^2^ g^−1^)	Pore volume (cm^3^ g^−1^)	Pore size (nm)
FZ0	142.25	0.310	9.0
FZ2	142.19	0.296	8.0
FZ4	163.19	0.276	6.8
FZ5	165.10	0.281	6.8
FZ6	228.11	0.225	4.2
FZ8	181.41	0.243	5.4
FZ10	101.12	0.137	5.5

**Fig. 6:**
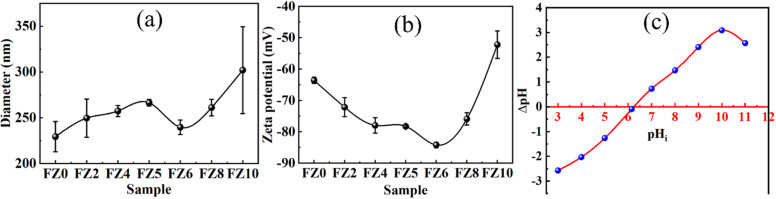
(a) Mean hydrodynamic diameter of Fe_1−*x*_Zn_*x*_Fe_2_O_4_ samples obtained from the dominant peak in the DLS distributions; (b) zeta potentials of the samples; and (c) point of zero charge (pH_i_) of FZ6.

The full DLS intensity distributions of the samples are provided in Fig. S1. The mean hydrodynamic diameters in Fig. 6(a) were extracted from the dominant peak in each distribution to ensure consistent comparison, since FZ0 exhibited a secondary peak associated with a minor agglomerated fraction.

Fig. 6(a) shows the hydrodynamic particle sizes of the Fe_1−*x*_Zn_*x*_Fe_2_O_4_ samples measured in deionized water. All samples consist of nanoscale primary crystallites but form secondary aggregates in dispersion due to magnetic dipole–dipole interactions. Among the series, the FZ6 sample displays a relatively narrow and symmetric size distribution, whereas FZ0 exhibits a bimodal profile and FZ10 shows a broader distribution associated with partial agglomeration. The mean hydrodynamic diameter of FZ6 (239.7 nm) therefore reflects a more stable dispersion state compared to the other compositions.

The zeta potential values in Fig. 6(b) further supports this observation. All samples possess negative surface charge, consistent with the presence of deprotonated surface –OH groups. However, the magnitude of the negative potential reaches a maximum in FZ6 (−85.2 mV), indicating stronger electrostatic repulsion between particles and enhanced colloidal stability. Although the absolute zeta potential is expected to shift under the acidic conditions used for adsorption (pH 3), the relative trend in surface charge among the samples remains unchanged and thus remains mechanistically meaningful.

Overall, although the BET surface area increases with Zn substitution and reaches its maximum for FZ6 (Table 1), the choice of FZ6 as the representative sample is further supported by its physicochemical dispersion behavior. As seen in Fig. 6, FZ6 exhibits a relatively narrow hydrodynamic size distribution and the most negative zeta potential (−85.2 mV), indicating superior colloidal stability compared to the other samples. The combination of large accessible surface area, reduced agglomeration, and strong negative surface charge suggests that FZ6 provides more effective active sites and enhanced interaction with aqueous pollutants. Therefore, FZ6 was selected for the subsequent adsorption and mechanistic studies.

The ΔpH − pHi profile (Fig. 6(c)) gives a point of zero charge of 6.3 ± 0.1. Accordingly, the surface of FZ6 is positively charged below pH of 6.3 and negatively charged above this value. This explains the markedly higher uptake of anionic species, HCrO_4_^−^/Cr_2_O_7_^2−^ for Cr(vi) and sulfonated DR79^−^, under mildly acidic conditions *via* electrostatic attraction and specific interaction with surface ≡M–OH_2_^+^ sites. When pH exceeds pH_i_, deprotonation (≡M–O^−^) leads to electrostatic repulsion and a pronounced drop in capacity; residual adsorption is attributed to hydrogen bonding and π–π interactions. The speciation shift of Cr(vi) (p*K*_a_ of 6.5) from HCrO4^−^ to CrO_4_^2−^ above pH of 6.3 further strengthens this trend.

### 3.2. Adsorption of DR79 and Cr(vi) on FZ6: kinetics, isotherms and thermodynamics

#### 3.2.1. Adsorption characteristics of the material toward DR79

##### 3.2.1.1 Effect of pH

**Fig. 7:**
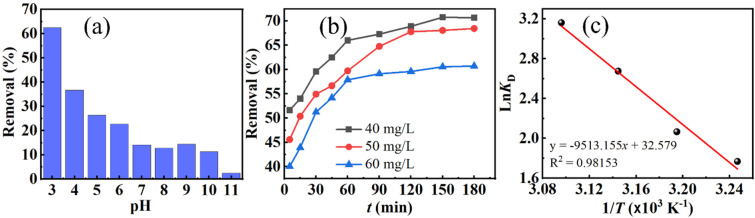
(a) Effect of pH on the adsorption efficiency of FZ6 toward DR79 (*m* = 0.020 g in *V* = 25 L, *C*_0_ = 50 mg L^−1^); (b) time-dependent removal at initial DR79 concentrations of 40, 50, and 60 mg L^−1^; and (c) van't Hoff plot ln *K*_D_*versus* 1/*T* (*T* in K).

Fig. 7(a) demonstrates that DR79 removal by FZ6 is strongly pH-dependent (adsorbent mass *m* = 0.020 g; solution volume *V* = 25 mL; initial dye concentration *C*_0_ = 50 mg L^−1^). The removal decreased monotonically with increasing pH: about 62.51% of the dye was adsorbed at pH values of 3, while only 2.45% remained at pH values of 11. This trend is consistent with the surface charge of FZ6 and the anionic nature of DR79. The point of zero charge (pHi = 6.26) indicates that the FZ6 surface is positively charged at pH < 6.26 and negatively charged at pH > 6.26.

At pH values of 3, extensive protonation maximizes the density of ≡M–OH_2_^+^ sites, which electrostatically attract the sulfonated dye anions DR79^−^, leading to high uptake. When pH values over 6.26, deprotonation produces ≡M–O^−^ sites; electrostatic repulsion dominates, and the adsorption efficiency drops sharply. In addition, excess OH^−^ at high pH competes with dye anions for surface sites.^26,27^ These observations agree with Dai *et al.*,^28^ who reported that cationic dyes are favored at high pH whereas anionic dyes are favored at low pH values. A similar pH-dependence has been reported for DR79 adsorption on FZ6. Based on these results, pH values of 3 were selected as the optimal pH for subsequent DR79 adsorption experiments on FZ6.

##### 3.2.1.2 Effect of contact time

Fig. 7(b) indicates a rapid increase in removal from 5 to 60 min, followed by a slower rise between 60 and 120 min; beyond 120 min, the removal becomes nearly constant (adsorbent mass *m* = 0.020 g; solution volume *V* = 25 mL; initial dye concentration *C*_0_ = 40; 50; 60 mg L^−1^). Thus, the equilibrium contact time for DR79 on FZ6 is taken as 120 min. The initial fast stage can be ascribed to abundant accessible sites on external surfaces, while the later stage reflects intraparticle diffusion and gradual occupation of energetically less favorable sites.

##### 3.2.1.3 Thermodynamics

The distribution coefficient was evaluated as 
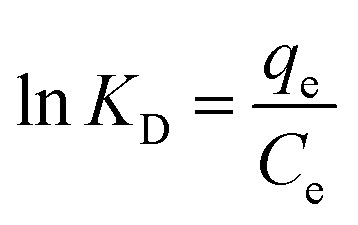
, a van't Hoff analysis (Fig. 7(c) and Table 2) using
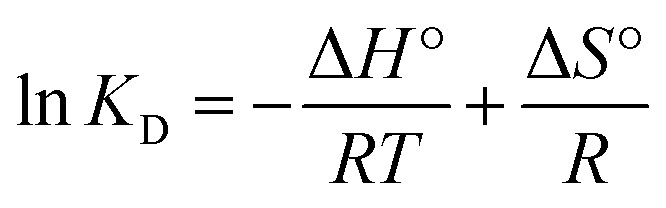
together withΔ*G*° = −*RT* ln *K*_D_ = Δ*H*° − *T*Δ*S*°

**Table 2:** Thermodynamic parameters (Δ*G*°, Δ*H*°, Δ*S*°) for DR79 adsorption onto FZ6 (derived from van't Hoff analysis)

*T*° (K)	1/*T*° (K^−1^)	*C*_e_ (mg L^−1^)	*q*_e_ (mg g^−1^)	*K*_D_	Δ*G*° (kJ mol^−1^)	Δ*H*° (kJ mol^−1^)	Δ*S*° (kJ mol^−1^ K^−1^)
308	0.003247	14.064	82.420	5.860	−4.528	79.092	0.271
313	0.003195	10.941	86.323	7.890	−5.375		
318	0.003145	6.344	92.070	14.514	−7.073		
323	0.003096	4.025	94.969	23.596	−8.489		

The parameters determined at the investigated temperatures are summarized in Table 2.

Linear regression of the van't Hoff plot (ln *K*_D_*vs. t*/*T*) yields Δ*H*°, and Δ*S*°, consequently, Δ*G*°, −*RT* ln *K*_D_ ranges from −4.528 to −8.489 kJ mol^−1^, confirming spontaneity and increasing favorability with temperature. The positive enthalpy changes Δ*H*° values of 79.092 kJ mol^−1^, indicates an endothermic process; increasing temperature enhances dye uptake, which is commonly associated with activation of chemisorptive interactions and/or reduced hydration of dye anions at the solid–liquid interface. The positive entropy changes Δ*S*° values of 0.271 kJ mol^−1^ K^−1^, suggests an increase in randomness during adsorption, plausibly due to the release of structured water molecules from the hydrated dye and surface sites upon complexation.

Combining the pH, kinetic, and thermodynamic results, DR79 removal by FZ6 proceeds most efficiently under mildly acidic conditions (pH values of 3) with an equilibrium contact time of around 120 min and is favored at elevated temperatures. The electrostatic attraction between protonated ferrite surfaces and sulfonated dye anions is the primary driving force, while the magnitude of Δ*H*° points to a significant chemisorption contribution.

#### 3.2.2. Adsorption characteristics of the material toward Cr(vi)

##### 3.2.2.1 Effect of pH

**Fig. 8:**
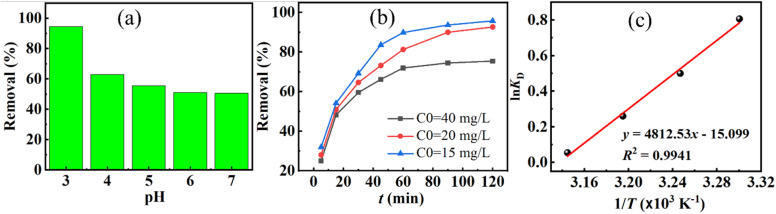
(a) Effect of pH on the removal efficiency of Cr(vi) by FZ6 (b) adsorption performance of FZ6 toward Cr(vi) at different initial concentrations and (c) van't Hoff plot showing the thermodynamic relationship between ln *K*_D_ and 1/*T* for Cr(vi) adsorption on FZ6.

Fig. 8(a) shows the influence of initial pH in range from 3 to7 on Cr(vi) removal by FZ6 at an initial concentration of 20 mg L^−1^ in 25 mL. Adsorption is highly dependent and decreases with increasing pH: 94.46% removal at pH values of 3, dropping to 62.83% at pH values of 4 (a decline of over 30 percentage points), and then decreasing more slowly to 50.56% at pH values of 7. This trend is explained by chromate speciation and the surface charge of FZ6. At low pH, Cr(vi) exists mainly as HCrO_4_^−^ and Cr_2_O_7_^2−^;^29^ electrostatic attraction between these anions and the positively charged (protonated) FZ6 surface promotes adsorption. As pH increases, surface deprotonation and the presence of OH^−^ give rise to (i) electrostatic repulsion between negatively charged FZ6 and chromate anions and (ii) competitive adsorption of OH^−^; jointly reducing uptake. Similar pH-dependent behavior for chromate has been reported by Kirankumar *et al.*^30^ Accordingly, pH values of 3 were selected as the optimal pH for subsequent experiments.

##### 3.2.2.2 Effect of contact time

Fig. 8(b) presents the time profiles at initial Cr(vi) concentrations of 15, 20, and 40 mg L^−1^ (25 mL solution; FZ6 dosage 0.020 g). All curves show a rapid increase in removal from 5 to 30 min (approximately linear), followed by a slower rise from 30 to 90 min, and an approach to a plateau between 90 and 120 min. The initial fast stage is attributed to the abundant accessible surface sites; the later stage reflects site saturation and intraparticle diffusion limitations. On this basis, the equilibrium contact time for Cr(vi) on FZ6 is taken as 90 min, which is used in subsequent studies.

##### 3.2.2.3 Thermodynamics

A van't Hoff analysis based on the linear relationship between ln *K*_D_ and 
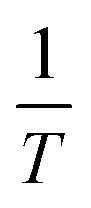
 (Fig. 8(c) and Table 3 yields thermodynamic parameters: the standard Gibbs free energy change Δ*G*° ranges from −2.032 to −0.146 kJ mol^−1^, indicating a spontaneous but weakly driven process; the enthalpy change is Δ*H*° values of −40.011 kJ mol^−1^, evidencing an exothermic adsorption, *i.e.*, lower temperatures favor uptake; and the entropy change is Δ*S*° values of −126 J mol^−1^ K^−1^, implying decreased randomness at the solid–liquid interface, likely due to the ordering/immobilization of chromate and interfacial water upon adsorption.

**Table 3:** Thermodynamic parameters (Δ*G*°, Δ*H*°, Δ*S*°) for DR79 adsorption onto FZ6 (derived from van't Hoff analysis)

*T* (K)	1/*T* (K^−1^)	*C*_e_ (mg L^−1^)	*q*_e_ (mg g^−1^)	*K*_D_	Δ*G*° (kJ mol^−1^)	Δ*H*° (kJ mol^−1^)	Δ*S*° (kJ mol^−1^ K^−1^)
303	0.00330	7.163	16.047	2.240	−2.032	−40.011	−0.126
308	0.00325	8.624	14.220	1.650	−1.281		
313	0.00320	9.813	12.734	1.298	−0.678		
318	0.00315	10.839	11.452	1.057	−0.146		

In summary, FZ6 removes Cr(vi) most effectively under mildly acidic conditions (pH values of 3) with an equilibrium contact time of around 90 min; adsorption is spontaneous, exothermic, and accompanied by an entropy decrease consistent with formation of ordered surface complexes.

#### 3.2.3. Adsorption kinetics and isothermal adsorption to DR79

**Fig. 9:**
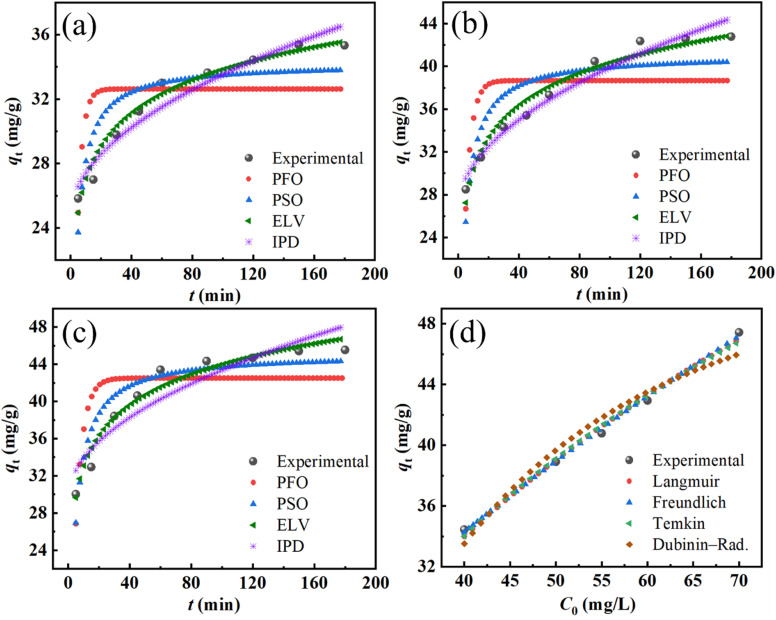
Nonlinear kinetic and isotherm fitting results for the adsorption of DR79 on FZ6: (a–c) kinetic modeling at different initial concentrations (40; 50; 60 mg L^−1^) using PFO, PSO, (ELV), and IPD models; (d) adsorption isotherm fitting with Langmuir, Freundlich, Temkin, and Dubinin–Radushkevich models at room temperature. Experimental data are denoted by solid symbols.

To gain insights into the adsorption mechanism of the synthesized material, four kinetic models were employed to fit the experimental data at different initial concentrations (*C*_0_ = 40, 50, and 60 mg L^−1^): pseudo-first-order (PFO), pseudo-second-order (PSO), Elovich, and intraparticle diffusion (IPD) models that shown in Fig. 9(a–c). The fitting quality was evaluated based on the correlation coefficient (*R*^2^), adjusted *R*^2^, and the reduced chi-square (*χ*^2^) value.

As summarized in SI – Table S2, the Elovich model exhibited the best fitting performance across all concentrations. For instance, at *C*_0_ of 40 mg L^−1^, the Elovich model yielded a reduced *χ*^2^ value of 0.452, *R*^2^ = 0.9684, and an adjusted *R*^2^ = 0.9639, significantly outperforming other models. A similar trend was observed for *C*_0_ of 50 mg L^−1^ and 60 mg L^−1^, where the Elovich model consistently showed the lowest reduced *χ*^2^ (1.023 and 1.476, respectively) and the highest *R*^2^ values (0.9670 and 0.9605, respectively).

These results indicate that the Elovich model provides the most accurate description of the adsorption kinetics. The suitability of this model suggests that the adsorption process involves heterogeneous surface binding and chemisorption, which is consistent with the presence of energetically non-uniform sites on the composite surface. Moreover, the poor fitting of the PFO and IPD models, particularly their relatively high *χ*^2^ and low *R*^2^ values, further confirms that the adsorption process does not follow simple diffusion or first-order kinetics.

Therefore, the Elovich model can be considered the most appropriate kinetic model for describing the adsorption behavior of the material under the tested conditions.

To evaluate the adsorption behavior of the material, four common isotherm models, including of Langmuir, Freundlich, Temkin, and Dubinin–Radushkevich (D–R), were fitted to the experimental data using nonlinear regression. The fitted curves are shown in Fig. 9(d), and the corresponding model parameters along with the goodness-of-fit indicators are summarized in Table 4. Among the models, the Freundlich isotherm provided the best fit to the data, with the highest coefficient of determination (*R*^2^ of 0.9991) and the lowest reduced chi-square value (0.013). This suggests that the adsorption occurred on a heterogeneous surface with multilayer coverage and varying adsorption energies. The Langmuir model also showed a strong correlation (*R*^2^ = 0.9916), indicating a significant monolayer adsorption component on the surface. The maximum adsorption capacity (*q*_max_) was estimated to be 95.3 mg g^−1^. The Temkin model (with a moderate *R*^2^ value of 0.9892), suggests that interactions between adsorbate molecules may be involved during the adsorption process. The Temkin constant *B* (22.87) also indicates moderate heat of adsorption.

**Table 4:** Nonlinear isotherm parameters for DR79 adsorption on FZ6 (Langmuir, Freundlich, Temkin, and Dubinin–Radushkevich models). Reported are the main model constants, *R*^2^, and *χ*^2^

Model	Main parameters	*R* ^2^	*χ* ^2^
Langmuir	*q*_max_ = 95.295, *K*_L_ = 0.01387	0.9916	0.259
Freundlich	*K*_F_ = 4.170, *n* = 1.751	0.9991	0.013
Temkin	*B*_T_ = 22.87, *K*_T_ = 0.111	0.9892	0.334
D–R	*q*_max_ = 53.757, *β* = 0.31275	0.9474	1.620

In contrast, the D–R model showed the poorest fit (*R*^2^ values of 0.9474 and *χ*^2^ values of 1.620), indicating that pore-filling mechanisms were less dominant in this adsorption process.

Based on these results, the Freundlich and Langmuir models were found to be the most appropriate for describing the adsorption behavior, while the D–R and Temkin models provided limited predictive value.

#### 3.2.4. Adsorption kinetics and isothermal adsorption to Cr(vi)

**Fig. 10:**
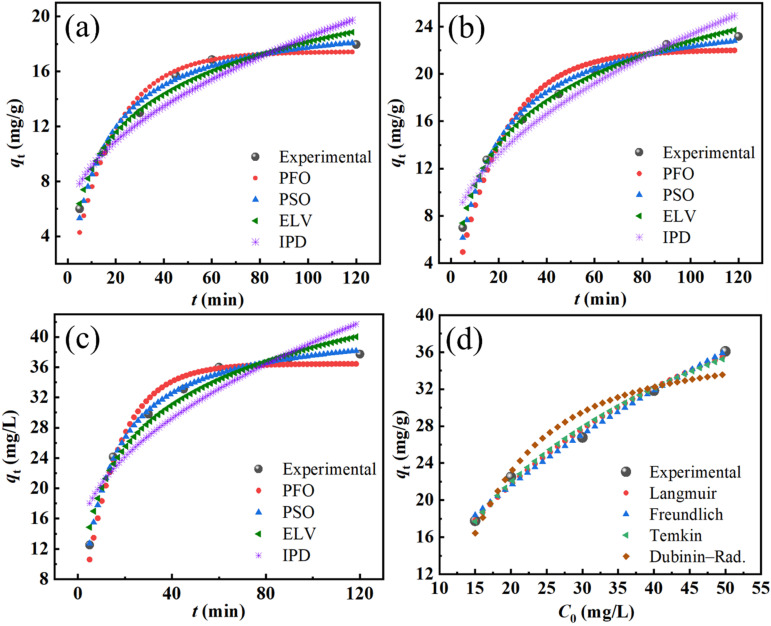
Nonlinear kinetic and isotherm fitting results for the adsorption of Cr(vi) on FZ6: (a–c) kinetic modeling at different initial concentrations (15; 20; 40 mg L^−1^) using PFO, PSO, (ELV), and IPD models; (d) adsorption isotherm fitting with Langmuir, Freundlich, Temkin, and Dubinin–Radushkevich models at room temperature. Experimental data are denoted by solid symbols.

The adsorption kinetics of Cr(vi) ions onto FZ6 were investigated using four nonlinear kinetic models, including pseudo-first-order (PFO), pseudo-second-order (PSO), Elovich, and intraparticle diffusion (IPD), as shown in Fig. 10(a–c). The corresponding kinetic parameters at three initial concentrations (15, 20, and 40 mg L^−1^) are summarized in SI – Table S3. Among the tested models, the PSO model exhibited the highest correlation coefficients at all concentrations (*R*^2^ = 0.9874–0.9970) and the lowest reduced *χ*^2^ values (0.298–0.463), indicating that the adsorption of Cr(vi) follows a chemisorption mechanism involving valence forces through sharing or exchange of electrons between adsorbent and adsorbate. The Elovich model also yielded relatively high *R*^2^ values (0.9642–0.9957), supporting the occurrence of heterogeneous surface adsorption. However, its reduced Chi-square values were slightly higher than those of the PSO model, especially at high concentrations (*e.g.*, 3.56 at 40 mg L^−1^), suggesting a less accurate fit under these conditions. The PFO model demonstrated moderate fitting, with *R*^2^ values ranging from 0.9450 to 0.9728, but significantly higher *χ*^2^ values (*e.g.*, 2.711 at 40 mg L^−1^), implying that it does not adequately describe the adsorption process compared to PSO or Elovich. The IPD plots did not pass through the origin and exhibited increasing interceptive values (*c* = 4.76–11.91), suggesting that intraparticle diffusion is involved in the adsorption process but is not the sole rate-limiting step. Moreover, the IPD model showed the lowest *R*^2^ values (0.8483–0.9401) and the highest *χ*^2^ values (up to 15.100), further confirming its limited role in describing the overall kinetics. Based on these results, the PSO model is considered the most appropriate to describe the adsorption kinetics of Cr(vi) on FZ6, highlighting the significance of chemical interaction between the metal ions and the adsorbent surface.

Langmuir, Freundlich, Temkin, and Dubinin–Radushkevich (D–R) models were investigated and are shown in Fig. 10(d) and Table 5. Among these models, the Freundlich model showed the highest correlation coefficient (*R*^2^ = 0.9938) and the lowest reduced *χ*^2^ value (0.437), suggesting that the adsorption process follows a heterogeneous multilayer mechanism. The adsorption intensity *n* = 1.786 further confirms a favorable adsorption process (since 1 < *n* < 10). The Langmuir model also provided a good fit, with *R*^2^ values of 0.99112 and *q*_max_ values of 62.063 mg g^−1^, indicating a relatively high adsorption capacity assuming monolayer coverage. However, its slightly higher chi-square value compared to Freundlich implies less agreement with the experimental data. The Temkin model yielded a lower correlation (*R*^2^ values of 0.9899), with *B*_T_ values of 14.675 and *K*_T_ values of 0.22272, indicating moderate interactions between adsorbate and adsorbent. The D–R model, on the other hand, showed the poorest fit (*R*^2^ values of 0.9245 and high *χ*^2^ values of 5.314), suggesting that its assumptions may not be suitable for describing the Cr(vi) adsorption onto FZ6 in this case. In summary, the adsorption of Cr(vi) on FZ6 is best described by the Freundlich isotherm, highlighting the heterogeneous nature of the surface and the possibility of multilayer adsorption.

**Table 5:** Nonlinear isotherm parameters for Cr(vi) adsorption on FZ6 (Langmuir, Freundlich, Temkin, and Dubinin–Radushkevich models). Reported are the main model constants, *χ*^2^ and reduced *R*^2^

Model	Main parameters	*χ* ^2^	*R* ^2^
Langmuir	*q*_max_ = 62.063, *K*_L_ = 0.02685	0.625	0.9911
Freundlich	*K*_F_ = 4.037, *n* = 1.786	0.437	0.9938
Temkin	*B*_T_ = 14.675, *K*_T_ = 0.22272	0.710	0.9899
D–R	*q*_max_ = 36.168, *β* = 0.07641	5.314	0.9245

As summarized in Table 6, FZ6 delivers a Langmuir capacity of 95.30 mg g^−1^ for DR79 at pH values of 3 (dose 0.8 g L^−1^, *t* = 120 min, *C*_0_ = 50 mg L^−1^). This value exceeds that of Fe_1−*x*_Co_*x*_Fe_2_O_4_ (56.85 mg g^−1^)^1^ and is slightly higher than a Fe_3_O_4_/CeO_2_ nanocomposite (90.5 mg g^−1^)^31^ under similarly acidic conditions. It is 2–10 time greater than low-cost biosorbents (palm-tree waste 9.79 mg g^−1^; wastewater sludge 13.4 mg g^−1^; coffee husk 36.63 mg g^−1^ and also surpasses an iron-oxide/carbon composite (45.8 mg g^−1^).^10^ Considering its magnetic retrievability, FZ6 thus couples competitive capacity with facile separation-an advantage over many non-magnetic, low-cost sorbents (Table 7).

**Table 6:** Comparison of reported anionic dye adsorption on oxide/ferrite-based adsorbents and this study

Adsorbent	Adsorbate	pH	Dose (g L^−1^)	Time (min)	*C*_0_ (mg L^−1^)	*q*_max_ (mg g^−1^) or % removal	Ref.
Fe_1−*x*_Co_*x*_Fe_2_O_4_	DR79	3	0.8	120	50	56.85	Hau *et al.*^1^
Fe_3_O_4_/CeO_2_ nanocomposite	Acid Black 210	7	1.0	120	50	90.5	Gao *et al.*^31^
Iron oxide/carbon composite	Congo Red	7	0.2	—	10	45.8	Singh *et al.*^10^
CoFe_1.9_Sm_0.02_O_4_@CS-ECH	Orange II	6	2.0	180	50	209.3	Humelnicu *et al.*^32^
SF-B-CoNiAl	Eriochrome Black T	2	0.33	180	20	329.61	Elkhider *et al.*^33^
	Methyl Orange	4	—	180	20	219.56	
Barium hexaferrite nanoparticles	Congo Red	—	—	20	—	124.7	Mohammed *et al.*^34^
Co_0.5_Mn_0.5_Fe_2_O_4_ nanoparticles	Congo Red	2	2.5	120	150	58.3	Zhang *et al.*^35^
CoFe_2_O_4_ nanoparticles	Congo Red	4	0.3	130	50	13.88	Sidhaarth *et al.*^36^
FZ6	DR79	3	0.8	120	50	95.295	This study

**Table 7:** Reported Cr(vi) uptakes on magnetic oxides/ferrites *versus* this work

Adsorbent	pH	Dose (g L^−1^)	Time (min)	*C*_0_ of Cr(vi) (mg L^−1^)	*q*_max_ (mg g^−1^)	Ref.
Fe_3_O_4_/chitosan/polypyrrole (Fe_3_O_4_/CS/PPy)	2	0.5	12 h	100	193.23	Yin *et al.*^7^
(Fe_3_S_4_)-CTAB	2	0.75	60	100	330.03	Zhou *et al.*^37^
Fe_2_O_3_–MnO_2_–SnO_2_	2	2.5	90	50	69.2	Uddin *et al.*^8^
NiFe_2_O_4_@AC	2	0.1	720	150	72.62	Zhang *et al.*^38^
NiFe_2_O_4_	3	0.2	55	20	294.1	Zandipak *et al.*^39^
MnFe_2_O_4_	3	3	480	10	5.813	Lu *et al.*^9^
Mg_0.2_Zn_0.8_Fe_2_O_4_					30.49	Tatarchuk *et al.*^40^
Fe_3_O_4_	4	2	90	0.001	8.67	Zhang *et al.*^41^
Magnetic nanobiosorbent from *Aspergillus niger* biomass	5.8	3.72	11	23.4	92%	Daneshvar *et al.*^42^
NiFe_2_O_4_ reflux synthesized	3	2	120	25	65%	Padmavathy *et al.*^43^
FZ6	3	0.8	90	20	62.063	This study

For Cr(vi), FZ6 achieves a Langmuir *q*_max_ of 62.06 mg g^−1^ at pH values of 3 (dose 0.8 g L^−1^, *t* = 90 min, *C*_0_ = 20 mg L^−1^). This performance is comparable to multi-oxide Fe_2_O_3_–MnO_2_–SnO_2_ (69.2 mg g^−1^) and NiFe_2_O_4_@AC (72.62 mg g^−1^),^8^ and significantly higher than MnFe_2_O_4_ (5.813 mg g^−1^),^9^ Fe_3_O_4_ (8.67 mg g^−1^),^41^ and Mg_0.2_Zn_0.8_Fe_2_O_4_ (30.49 mg g^−1^).^40^ Although some specialized adsorbents report much larger capacities such as (Fe_3_S_4_)-CTAB (330.03 mg g^−1^)^37^ or NiFe_2_O_4_ (294.1 mg g^−1^),^39^ these frequently require harsher acidity (pH values of 2), different doses, or long contact times (*e.g.*, 720 min for NiFe_2_O_4_@AC.^38^ In contrast, FZ6 offers a balanced profile: moderate-to-high capacity at pH values of 3, short equilibrium time (∼90 min), and magnetic separability, while simultaneously functioning as a dual adsorbent for both an anionic dye and a metal oxyanion.

## 4. Conclusion

The Zn-substituted ferrite nanoparticles (Fe_1−*x*_Zn_*x*_Fe_2_O_4_) were an effective and magnetically separable platform for the dual removal of DR79 and Cr(vi) from water. The materials possess nanometric spinel crystallites, mesoporosity, and composition-dependent magnetization; among them, FZ6 combines the largest surface area with robust adsorption performance. Electrostatic attraction dominates the uptake when the pH is below the pH_i_ value of 6.26, with the best removal efficiency obtained at pH 3. The adsorption process reached equilibrium after about 120 min for DR79 and 90 min for Cr(vi). Kinetic analysis using nonlinear fitting shows that DR79 follows the Elovich model, whereas Cr(vi) is better described by the pseudo-second-order model. For equilibrium studies, the nonlinear Freundlich model provides the best fit across the tested concentration ranges. The maximum adsorption capacities calculated from the Langmuir model were about 95.3 mg g^−1^ for DR79 and 62.1 mg g^−1^ for Cr(vi), indicating good adsorption performance. Thermodynamic evaluation further points to different mechanisms: DR79 adsorption is endothermic with a positive entropy change, suggesting increased disorder at the solid–liquid interface, while Cr(vi) adsorption is exothermic with a negative entropy change, implying a more ordered interfacial structure. These differences confirm that both systems proceed through chemisorption, but with distinct interfacial arrangements. Taken together, these findings position Fe_1−*x*_Zn_*x*_Fe_2_O_4_, particularly FZ6 as a versatile, recyclable adsorbent for integrated treatment of mixed dye/metal-bearing effluents. Future work should prioritize regeneration/long-cycle stability, competitive adsorption in multisolute matrices, ionic-strength effects, and spectroscopic probes (*e.g.*, XPS) to resolve any redox contributions during chromate sequestration.

## Conflicts of interest

The authors declare that they have no conflict of interest.

## Supplementary Material

RA-015-D5RA07081C-s001

## Data Availability

The datasets produced and analyzed during this study are available from the corresponding author upon reasonable request. Supplementary information (SI): additional adsorption data, fitting plots, characterization results, and experimental details. See DOI: https://doi.org/10.1039/d5ra07081c.
