# Supplementary material for: Magnetic Fe1−xZnxFe2O4 nanoparticles as dual adsorbents for Cr(vi) and Direct Red 79: kinetics, isotherms, and mechanistic insights
Source: RSC Adv. 2025 Dec 17;15(59):50795–809. doi: 10.1039/d5ra07081c (PMC12709496; doi:10.1039/d5ra07081c)
Supplement: RA-015-D5RA07081C-s001 [file RA-015-D5RA07081C-s001.pdf]

Supplementary Information (SI) for RSC Advances.
This journal is © The Royal Society of Chemistry 2025

Magnetic $Fe_{1-x}Zn_xFe_2O_4$ Nanoparticles as Dual Adsorbents for Cr(VI) and Direct Red 79: Kinetics, Isotherms, and Mechanistic Insights

Vu Thi Hau[1], Nguyen Thuy Chinh[2], Pham Hoai Linh[2], Nguyen Thi To Loan[1], Ngo Thi Mai Viet[1], Dang Duc Dung[3], Nguyen Quoc Dung[1*]

[1]Faculty of Chemistry, Thai Nguyen University of Education,

20 Luong Ngoc Quyen, Thai Nguyen, Vietnam

[2]Institute of Materials Science, Vietnam Academy of Science and Technology,

18 Hoang Quoc Viet, Nghia Do, Ha Noi, Viet Nam

[3)]Multifunctional Ferroics Materials Lab., Faculty of Engineering Physics,
Ha Noi University of Science and Technology, 1 Dai Co Viet road, Ha Noi, Viet Nam

Corresponding author: Nguyen Quoc Dung

E-mail address: dungnq@tnue.edu.vn

3. Results and discussion

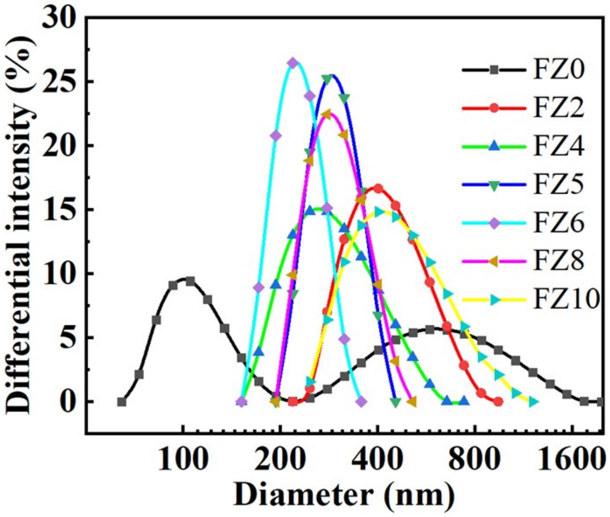

Figure S1. Full DLS differential intensity distribution curves.

Table S1. Reagent composition for material preparation.

x	2 M $FeCl_2$ (mL)	2 M $FeCl_3$ (mL)	2 M $ZnCl_2$ (mL)	2 M HCl (mL)	2 M NaOH (mL)	Denotation of product
0	2	4	0	15	60	F0
0.2	1.6	4	0.4	15	60	FC1
0.4	1.2	4	0.8	15	60	FC2
0.5	1.0	4	1.0	15	60	FC3
0.6	0.8	4	1.2	15	60	FC4
0.8	0.4	4	1.6	15	60	FC5
1	0	4	2	15	60	C0

Table S2. Nonlinear kinetic parameters for DR79 adsorption on FZ6 at C_0 = 40-60 mg/L (models: PFO, PSO, Elovich, IPD). Reported: q_e, k_1, k_2, α, β, k_{id}, c, reduced χ^2, and R^2.

**Model**	C_0 **(mg/L)**	q_e	k_1	k_2	α	β	k_{id}	c	χ^2	R^2
PFO	40	32.630	0.28925						8.172	0.4298
PSO	40	34.218		0.01322					3.497	0.7560
Elovich	40				2632.442	0.3367			0.452	0.9684
IPD	40						0.89745	24.550	0.906	0.9368
PFO	50	38.675	0.23414						17.06	0.4499
PSO	50	41.102		0.0079					7.695	0.7519
Elovich	50				445.693	0.2289			1.023	0.9670
IPD	50						1.34015	26.509	1.079	0.9652
PFO	60	42.510	0.19961						15.927	0.5737
PSO	60	45.165		0.00655					5.164	0.8618
Elovich	60				474.450	0.2093			1.476	0.9605
IPD	60						1.39037	29.462	5.140	0.8624

Table S3. Nonlinear kinetic parameters for Cr(VI) adsorption on FZ6 at C_0 = 15-40 mg/L (models: PFO, PSO, Elovich, IPD). Reported: q_e, k_1, k_2, α, β, k_{id}, c, reduced χ^2, and R^2.

**Model**	C_0 **(mg/L)**	q_e	k_1	k_2	α	β	k_{id}	c	χ^2	R^2
PFO	15	17.436	0.05637						1.005	0.9578
PSO	15	20.211		0.00354					0.301	0.9874
Elovich	15				2.952	0.2344			0.527	0.9778
IPD	15						1.3756	4.7558	2.472	0.8961
PFO	20	22.047	0.05082						2.196	0.9450
PSO	20	25.871		0.00241					0.463	0.9884
Elovich	20				3.030	0.1752			0.171	0.9957
IPD	20						1.8244	5.0617	2.392	0.9401
PFO	40	36.448	0.06867						2.711	0.9728
PSO	40	41.832		0.00209					0.298	0.9970
Elovich	40				8.154	0.1187			3.560	0.9642
IPD	40						2.7371	11.9087	15.100	0.8483
